# Uronium from X‑ray-Desorbed Urea Enables Sustainable Ultrasensitive Detection of Amines and Semivolatiles

**DOI:** 10.1021/acs.analchem.5c02239

**Published:** 2025-09-28

**Authors:** Aleksei Shcherbinin, Henning Finkenzeller, Fariba Partovi, Netta Vinkvist, Jussi Kontro, Matthew Boyer, Joona Mikkilä, Siddharth Iyer, Jyri Mikkilä, Paxton Juuti, Nina Sarnela, Juha Kangasluoma, Matti Rissanen

**Affiliations:** † Karsa Ltd., Helsinki 00560, Finland; ‡ Institute for Atmospheric and Earth System Research/Department of Physics, Faculty of Science, 610542 University of Helsinki, Helsinki 00014, Finland; § Aerosol Physics Laboratory, Physics Unit, Faculty of Engineering and Natural Sciences, 7840 Tampere University, Tampere 33014, Finland; ∥ Department of Chemistry, Faculty of Science, University of Helsinki, Helsinki 00014, Finland

## Abstract

Comprehensive mass spectrometric detection requires multiple ionization schemes. Chemical ionization (CI) at a low pressure is suitable for the detection of weakly polar volatile organic compounds (VOCs). Negative-mode ionization at ambient pressure delivers a superior performance for polar acidic compounds. Positive-mode CI has been explored to detect basic and polar neutral compounds for which negative polarity and low-pressure ionization techniques have shown insufficient performance. Several ion attachment reagents have been proposed for sensitive and soft ionization. These reagents are often reactive, toxic, and difficult to control, which impede their applicability and operability. Inspired by these challenges, we explored uronium, protonated urea, as an alternative for ionizing moderately oxygenated, basic, and polar neutral compounds at ambient pressure. Urea, a nontoxic solid with negligible vapor pressure, is desorbed by X-ray irradiation, forming the uronium ion. We experimentally determined the behavior of uronium ionization under different humidities for several semivolatile organic compounds (SVOCs), amines, and ammonia and explored the mechanism using theory. In laboratory measurements of α-pinene and dimethyl sulfide (DMS) oxidation systems, we characterized how uronium complements other ionization schemes. Excellent sensitivities were achieved for several key components (including amines, dimethyl sulfoxide (DMSO), *N*-methyl-2-pyrrolidone, verbenone, and dimethylformamide (DMF)), requiring sample sizes of only a few attomoles for detection in individual spectra, equivalent to detection limits at the low to mid parts per quadrillion by volume (ppqv) level. Uronium exhibits a tendency to selectively form strong ion–molecular clusters, which renders the ionization robust against sample humidity changes. X-ray desorption of solid urea simplifies reagent supply handling and ensures the long-term stability of the ion production system, providing a safe and sustainable alternative to equivalent CI methods.

## Graphical abstract

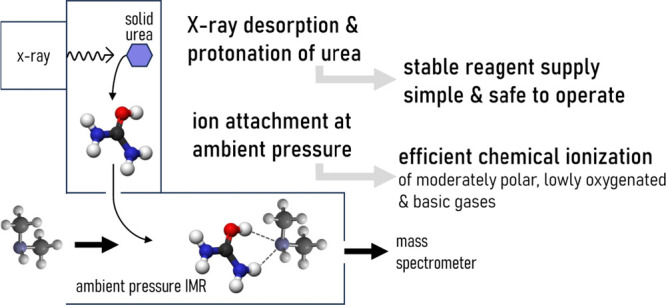

## Introduction

Chemical ionization mass spectrometry (CIMS) is an essential analytical technique for the direct gas-phase analysis of a wide range of compounds in multiple applications including industrial process control,^1^ air quality monitoring,^2^ food science,^3^ medical research,^4^ and atmospheric science.^5^ Multiple techniques have been developed to efficiently detect a wide range of molecules efficiently. The polarity,^6^ pressure,^7^ temperature,^8,9^ and electronic structural features^10^ of the reagent ions define the dimensionality of the chemical ionization (CI) regimes, resulting in a specific selectivity and sensitivity profile to particular compound groups.

Proton-transfer-reaction mass spectrometry (PTR-MS) using hydronium as a reagent ion at low pressure has become a widely adopted technique for analyzing volatile organic compounds (VOCs).^11^ While it is an excellent analysis tool for less polar and more volatile molecules, it is less selective and more fragmenting toward functionalized species.^12–14^ Polar acidic and highly oxygenated species are best detected by negative-mode CI, using, e.g., nitrate, bromide, or iodide.^15–18^ Several alternative techniques have been proposed for the sensitive detection of semivolatile organic compounds (SVOCs), which are often less oxygenated, moderately polar, and basic molecules, for which PTR-MS and negative-mode ion attachment methods exhibit reduced sensitivity. The most notable methods are ammonium^19^ and aminium^6,20^ CIMS. While the methods are versatile and sensitive, these reagents are reactive, hazardous, and difficult to control. Müller et al.^21^ developed a novel low-pressure ammonium generation method that does not require the provision of ammonia, reducing the risks related to corrosion and toxicity. Still, the method does not fully meet the requirements for accurate and sensitive measurements. One of the limiting factors for the ammonium ionization is the low mass of the primary ion, which presents challenges related to ion transmission in the mass spectrometer, limiting the control and quantification capabilities of the method.^22^ In addition, the common method of providing ammonia in an aqueous solution is prone to drift, affecting the primary ion distribution and consequently altering the ionization regime.^19,22^ Despite these limitations, ammonium garnered significant interest due to the ability of the ion to cluster with a wide range of compounds, providing capabilities complementary to conventional PTR-MS and negative-mode CIMS. Driven by these challenges and the goal of thoroughly analyzing gas-phase species, we sought a better method for positive-mode ion attachment ionization targeting SVOCs, in particular moderately oxygenated, basic, and polar neutral compounds. The results of this exploration are the ionization by uronium, i.e., protonated urea, and X-ray desorption of solids for reagent ion production.

Urea (NH_2_C­(O)­NH_2_) is a simple yet remarkable organic compound known for its biological, chemical, and industrial relevance. Urea became the first organic compound synthesized from inorganic substances by Friedrich Wöhler^23^ in 1828, challenging the vitalism theory of organic chemistry and marking the advent of modern organic synthesis.^24^ Urea is a well-known hydrogen bond donor that forms strong eutectic mixtures with a variety of compounds.^25^ Structurally, urea is composed of two amino groups (−NH_2_) attached to a central carbonyl group (C=O)—a configuration that imparts polarity and the ability to form hydrogen bonds. This molecular arrangement renders urea highly soluble in water, facilitating its natural occurrence in biological systems as the principal nitrogenous waste product of protein metabolism. Synthesized in the liver, it provides a nontoxic mechanism for ammonia excretion, underscoring its critical role in maintaining nitrogen balance in living organisms.^26^

To the best of our knowledge and notwithstanding its emblematic role in science and favorable electronic structure, urea has never been tested as a CIMS reagent. Given its low vapor pressure, the main challenge to overcome in this study was to reliably produce gaseous uronium.

In this study, we found that X-ray desorption of solid urea at room temperature enables the generation of an intense, stable beam of uronium cations that can be sustained over long periods without maintenance. Using quantum-chemical computations, we explored the energetics and mechanism of ionization of neutral molecules by uronium. Next, we experimentally determined the sensitivity of uronium ionization to a spectrum of chemical compounds over a broad humidity range. To demonstrate the applicability of the method for the quantification of ammonia in ambient air, we performed a side-by-side comparison with a dedicated quantum cascade tunable infrared laser differential absorption spectrometer (QC-TILDAS, Aerodyne Research Inc.).^27^ Finally, using uronium CI in multipressure CIMS (MPCIMS),^7^ we explored its complementarity to other CI reagent ions in VOC oxidation experiments, which yield a wide spectrum of chemically diverse products.

## Methods

### Instrumentation

This study uses a multischeme CI inlet 2 (MION2, Karsa Ltd.) operating at ambient pressure^28,29^ coupled to an Orbitrap Exploris 120 mass spectrometer (Thermo Fisher Scientific Inc.)^30^ with a nominal mass resolution of 120,000. In brief, the MION2 inlet generates ions off-axis in a dedicated volume. Here, we introduce a novel method for the generation of ions using X-ray desorption of solids (patent application submitted).

Figure 1 shows the geometry of how solid urea was provided for the ionization volume. High-purity urea (VWR Chemicals, analytical reagent grade) is deposited into a custom-made holder (polyether ether ketone), which is placed between MION2 electrodes, exposing it to X-ray. The physical process of how urea is understood to sublimate is discussed in the Results and Discussion section. An electric field transports the uronium ions into the ambient-pressure ion–molecule reactor (IMR). The reagent gas in the ionization volume is kept apart from the sample gas by auxiliary gas flows.^29^ The reaction time within the IMR is approximately 25 ms.^29^ The sample gas then enters the Orbitrap mass spectrometer through a capillary (inner diameter 0.58 mm, length 58 mm) heated to 300 °C. Data were analyzed with the Orbitool software package.^31^ For VOC oxidation experiments, the ionization schemes were cycled between uronium cations (NH_2_C­(O)­NH_3_^+^), nitrate anions (NO_3_^–^), and fluoranthenium radical cations (C_16_H_10_^+^)^7^ to assess the complete gas-phase product distribution.

**Figure 1:**
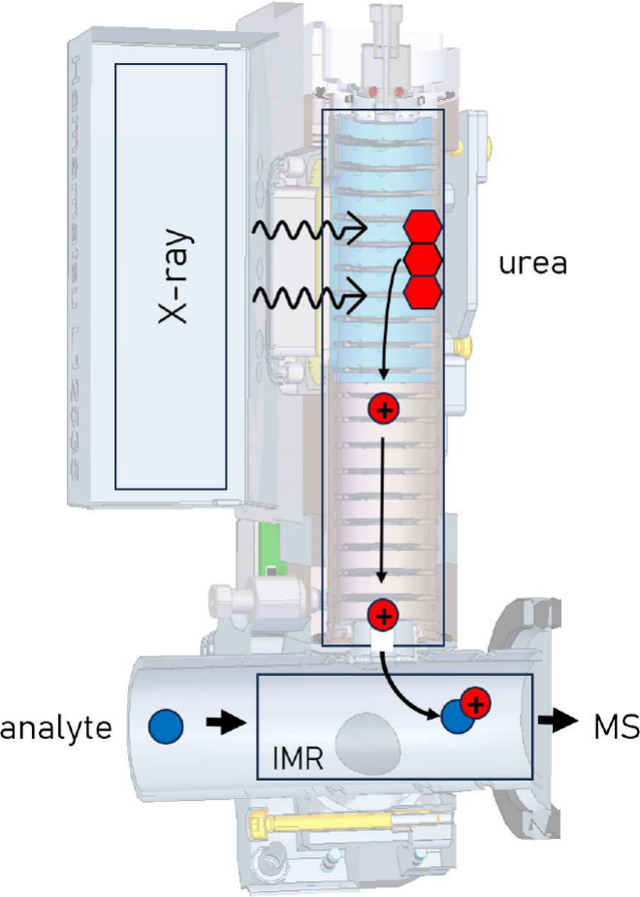
Setup for the uronium generation from solid urea by X-ray desorption and transfer of uronium into the ambient-pressure IMR of the MION2 inlet.^29^

The long-term stability of the ionization method was assessed on a TD-APCI-TOF system,^32^ where the ionization unit was updated with a MION2 uronium source. Independent ammonia measurements were performed using a QC-TILDAS instrument, which has been described elsewhere.^27,33–35^

### Quantum-Chemical Calculations

The energetics of uronium ionization, i.e., the formation enthalpies of analyte–uronium clusters and the energies associated with declustering into protonated analyte, were explored using computational quantum-chemical methods described in the SI.

### Measurement Data

This study generated data from multipoint calibrations at different humidities, as well as data from laboratory flow reactor experiments aiming at α-pinene and dimethyl sulfide (DMS) oxidation products, and observations from ambient field measurements.

The sensitivity of uronium for the detection of a number of commercially available compounds (Table S2) was determined in a set of calibration experiments that used a liquid calibration unit (Ionicon Analytik^36^). In the apparatus, dilute aqueous analyte solutions are sprayed (delivery rate 0.5–15 μL min^–1^) into a heated chamber (110 °C) and carried by a carrier flow (1 lpm) into the main sample flow (20 lpm). The respective concentration of the dilute solutions was iteratively determined to typically attain a normalized signal of a few percent. The mixing ratio of the introduced calibrant ranged from pptv to several ppbv, depending on the compound tested. Multipoint calibrations were performed with varying degrees of humidification, from dry (RH< 5%) to 80% relative humidity, and dosing levels varying by up to a factor of 30 (Figure S1).

To evaluate the quantification capabilities under ambient conditions, we performed a side-by-side comparison with a dedicated ammonia instrument (QC-TILDAS) that was temporarily available at the institute. Ambient air was sampled through a makeshift inlet (PTFE tubing with 4 m length and 10 mm inner diameter) from the window of a laboratory located in the suburbs of Helsinki, Finland. At the end of the ambient measurements, both instruments sampled indoor (laboratory) air for a few hours, during which ammonia was also evaporated from a cloth to explore the response of the instrument to higher concentrations.

To create a wide spectrum of compounds with a variable degree of oxidation, qualitative α-pinene oxidation experiments were carried out in a flow tube reactor (reaction time 6 s). α-Pinene vapors were obtained by bubbling nitrogen through a liquid reservoir. The α-pinene volume mixing ratio (VMR) in the flow tube reactor was approximately 4 ppmv. Ozone was generated from synthetic air by using UV light irradiation.

DMS oxidation experiments employed the same flow tube apparatus as that used in the α-pinene experiments. DMS was dosed from a bottle (Air Products, 100 ppmv). Ethylene was added as a dry OH source, and ozone was generated from synthetic air via UV light irradiation.

## Results and Discussion

### Uronium Generation

The saturation vapor pressure of urea is very low, reaching only about 10^–8^ atm at room temperature (Figure S5). Experimentally, uronium ions are observed only if some of the (solid) urea is illuminated by the X-ray charger (Figure 1)—providing urea vapors conventionally from a reservoir of solid urea in an external reagent vial does not create any uronium signal, not even if the length of lines is minimized and ample time (1 week) is allowed for conditioning. When urea is introduced into a clean MION2 ion source and exposed to X-ray, the uronium signal appears instantaneously.

The exact mechanism by which urea sublimates to the gas phase is not known; e.g., it is not clear whether neutral urea, uronium, or urea radicals evaporate. The enthalpy of sublimation of urea is approximately 95 kJ mol^–1^ or 1 eV.^37^ X-ray ionization ejects electrons from their orbital. Relaxation of the atom to a lower electronic state releases energy (typically multiple eV) for evaporation to occur.^38–41^ This is similar to matrix-assisted laser desorption/ionization (MALDI), although the exact mechanism of volatilization and charging remain unclear.^42^ We conclude that X-ray irradiation is required to induce significant sublimation of urea and the generation of uronium ions.^43,44^

We tested the approach to generate reagent ions from solids other than urea. The X-ray irradiation of ammonium nitrate salts also produces nitrate ions. Here, the combined abundance of the nitrate monomer and dimer is three times higher than that when the nitrate is formed from the irradiation of nitric acid vapors, provided from a liquid reservoir. The mechanism of ion production from a solid precursor substance appears to be not limited to urea and warrants further exploration.

Figure 2a shows the mass spectrum in the absence of analytes. Uronium and the urea–uronium cluster are the only dominant peaks. The observation of the dimer is similar to that of other ionization approaches (NO_3_^–^, Br^–^, etc.). The uronium–water peak was observed only at trace intensities at high RH (1000 cps or 10^–4^ ncps (normalized counts per second, i.e., unscaled ratio of analyte ion and reagent ion intensity) at 80% RH). This is consistent with a relatively low binding enthalpy between uronium and water (theoretical analysis given below). The abundance of contaminant peaks is low, with contaminants not exceeding 5 × 10^–3^ relative intensity (Figure 2a) when analytical-grade urea and zero-purity, high-purity air are used.

**Figure 2:**
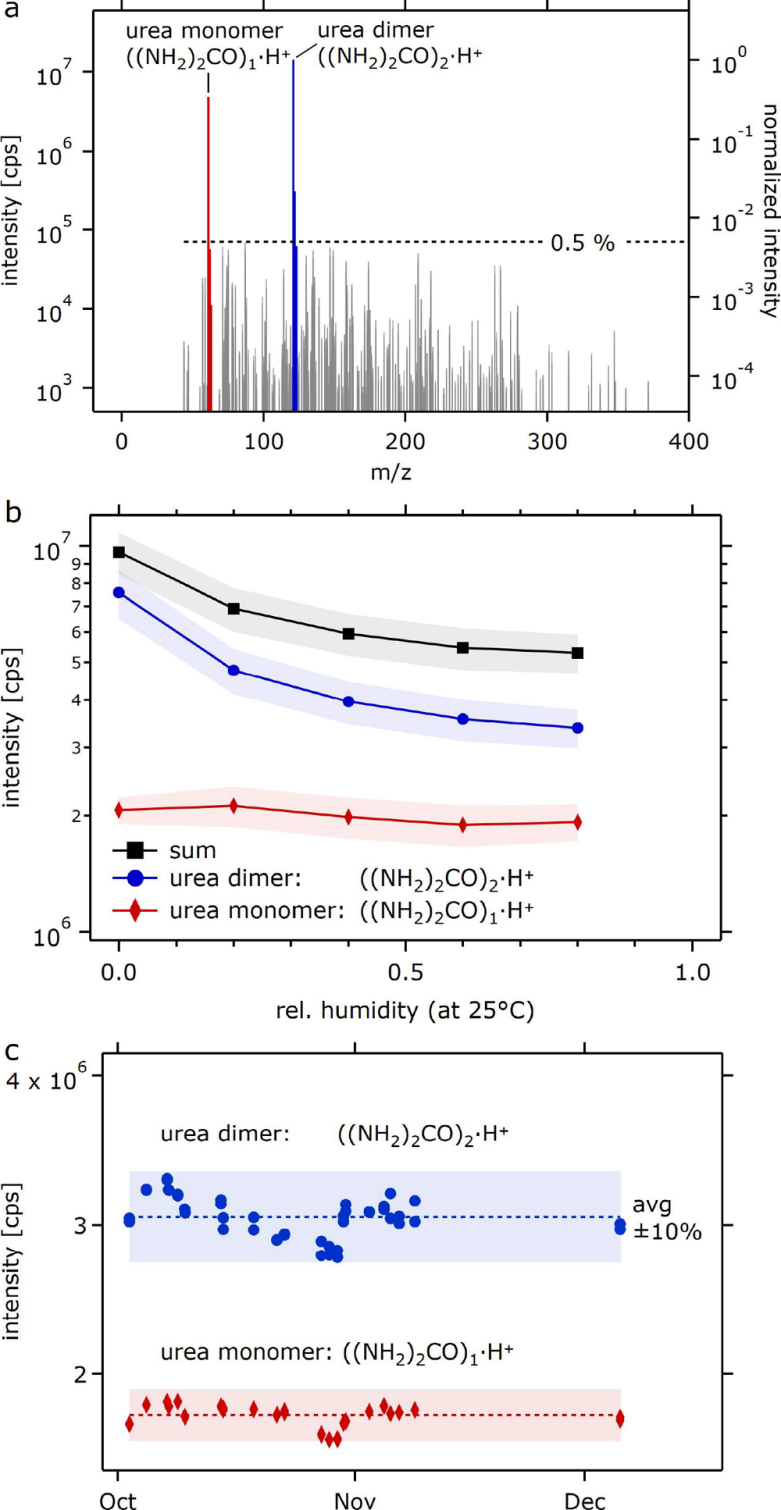
(a) Spectral purity of the uronium spectrum in the absence of an analyte (note the logarithmic intensity axis); (b) sensitivity of reagent ion intensities to humidity changes (shades indicate ± 1 standard deviation); and (c) the stability of reagent ion provision over 2 months. The long-term data in (c) were acquired on an instrument different from that used in (a) and (b).

The sensitivity of the abundance of reagent ions to changes in humidity is shown in Figure 2b. The abundance of the monomer is essentially constant. The abundance of the dimer decreases by approximately 50% when progressing from dry to wet conditions (80% RH). Between 20 and 80% RH, the decrease is only 20%. The exchange reaction

$$\mathrm{Ur}\cdot \mathrm{Ur}\cdot H^{+}+H_{2}O\to \mathrm{Ur}+\mathrm{Ur}\cdot H_{2}O\cdot H^{+}$$ 1

­(here, Ur denominates urea) is energetically not favorable (enthalpy difference 14.6 kcal mol^–1^) but still occurs due to the sheer abundance of water.

Figure 2c shows the remarkable long-term stability of uronium generation over more than 2 months. Without any retuning or resupply of the reagent, neither the monomer nor the dimer abundance deviated more than 10% from the average. The result is a testament to the low evaporation rate, even in the presence of constant X-ray irradiation. The robust operation without the need for daily or even monthly maintenance should not only facilitate easier deployments but—by introducing fewer changes—also improve the consistency and thereby overall quality of the acquired data.

### Ionization Mechanism

Figure 3 shows the theoretically determined cluster geometries for the selected compounds containing different functional groups. The associated binding enthalpies are shown in Figure S6 and tabulated in Table S1. The protonation of urea occurs preferentially at the carbonyl group, as the resulting cation is 16.02 kcal mol^–1^ lower in enthalpy compared to the cation with the proton on the nitrogen atom. The calculated proton affinity is 209.85 kcal mol^–1^ (compare literature value of 208.8 kcal mol^–1^ ^45^ and an estimated 1.6 kcal mol^–1^ error margin of the used DLPNO–CCSD­(T) method in predicting binding enthalpies^46^), and the dipole moment is 2.34 D; ammonium, in contrast, does not possess a net dipole moment. The energetically favorable cluster geometries for amines and ammonia have a single interaction with uronium, while the other studied molecules establish two or more interactions from the carbonyl and amino groups of uronium to form stable clusters. Analytes with multiple polar functional groups may exhibit interactions with multiple groups, e.g., triethylene glycol (Figure 3h), syringol, or glyoxal.

**Figure 3:**
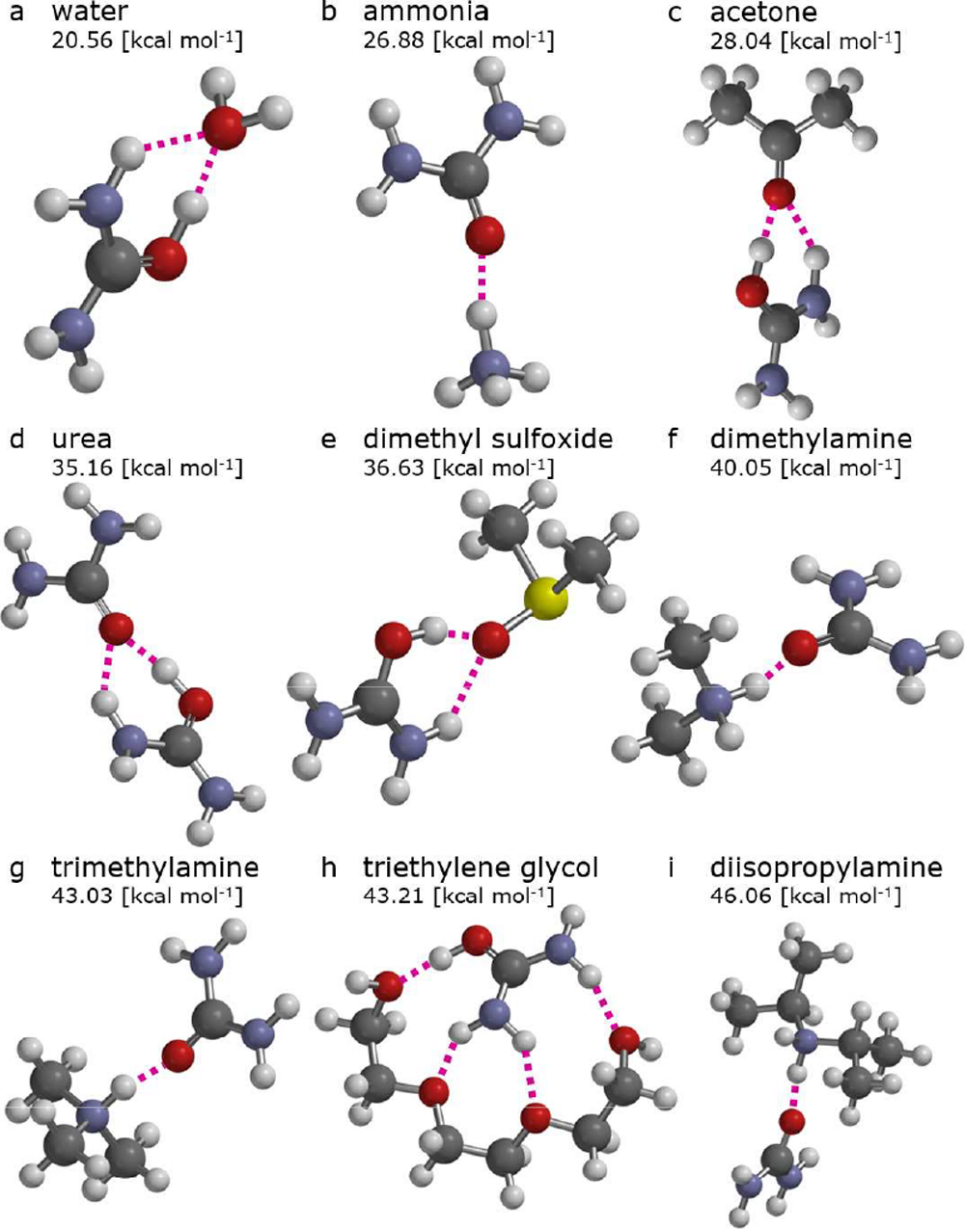
Geometries of clusters between uronium and the selected analytes.

The interaction of uronium connected to two functional groups distinguishes it from, for example, ammonium, where only one significant interaction is established. The affinities of uronium to form a cluster are generally slightly stronger than those of ammonium (Table S1 and Xu et al.)^22^, due to the higher electronegativity of oxygen compared to nitrogen. As a result, uronium clusters tend to be more stable.

Small molecular clusters with less than about 8 atoms are likely not efficiently collisionally stabilized during ionization, leading to lower instrument sensitivities despite strong binding enthalpies.^17,47^ For example, the CI of IO by bromide or nitrate is not as efficient as that of, e.g., sulfuric acid.^48^ The substantial number of atoms of uronium should make it a better reagent ion to detect small molecules such as IO. However, in the particular case of IO (and other iodine oxidation products), the calculated binding enthalpy was found to be unfavorably low (Table S1).

The enthalpies for different clusters against declustering into uronium and the analyte depend on the specific compound (Figure S6). The enthalpies were calculated, as they were shown to predict ionization efficiencies better than free energies.^47^ Higher binding enthalpies generally lead to higher chances of ionization, as long as the system size allows sufficient dissipation of energy.^17,47^

The enthalpy for uronium to cluster with urea to form the Ur ·Ur ·H^+^ dimer ion (35.16 kcal mol^–1^, Figure 3d) influences the extent to which the dimer participates in the formation of analyte–uronium clusters. Due to the strong binding enthalpy of the uronium–urea cluster, it forms stable reagent ion dimers under our experimental conditions. The ionization of an analyte then happens through a ligand-exchange reaction, where analyte–uronium clusters with binding enthalpies in excess of 35.16 kcal mol^–1^ are more efficiently ionized than the more weakly bound clusters. Importantly, the binding enthalpy between uronium and water is relatively low (20.56 kcal mol^–1^; Figure 3a), such that the reagent ion is not strongly interacting with water and humidity sensitivities are minor.

Another route for the newly formed uronium–analyte clusters is declustering into neutral urea and protonated analyte. This is more likely for compounds with a high proton affinity. The energetics of the uronium proton transfer reactions were determined for different compounds and are illustrated in Figure S7, showing trends and outliers within different compound classes. The extent to which declustering occurs depends on the energies, the distribution of energy within the cluster, and the dissipation of excess energy to the bath gas. The initial interaction between uronium and the analyte determines the overall likelihood for ionization.

The trend of increasing binding enthalpies for, e.g., the amines is the combined result of the changing dipole moment and the ability of the larger molecules to adopt energetically favorable configurations.

### Empirical Ionization Efficiencies

Figure 4 shows the measured calibration factors derived from the multipoint calibrations under varied humidity (see Methods) for the different compounds on the left axis, while the right axis indicates the respective limits of detection, estimated as the VMR corresponding to the assumed lowest detectable peak intensities of 100 cps. All signals are normalized by the sum of the uronium monomer and dimer ion. The abscissa represents the calculated binding enthalpy of the uronium–analyte cluster, which is the energy associated with the first interaction between the analyte and uronium. Markers indicate the calibration factor for the detection as a cluster (blue diamond) or a protonated analyte (red circle). The dashed gray lines connect the two respective experimental calibration factors via the binding enthalpy of the cluster against declustering into the protonated analyte and neutral urea. The uronium–analyte clusters tend to decluster into the protonated analyte and neutral urea if the process is energetically favorable. In this case, the detection of a target compound via the protonated analyte yields better sensitivity. For example, the declustering of the uronium–trimethylamine cluster into urea and trimethylaminium is favorable by about 15 kcal mol^–1^, and trimethylamine is mostly detected as trimethylaminium. Vice versa, triethylene glycol is mostly detected as a cluster with uronium. As expected from the theory, higher sensitivities are found for larger binding enthalpies.^47^ Acetone is detected with a surprisingly low sensitivity, which we speculate to originate from some sort of reactive interaction between acetone and urea.^49^

**Figure 4:**
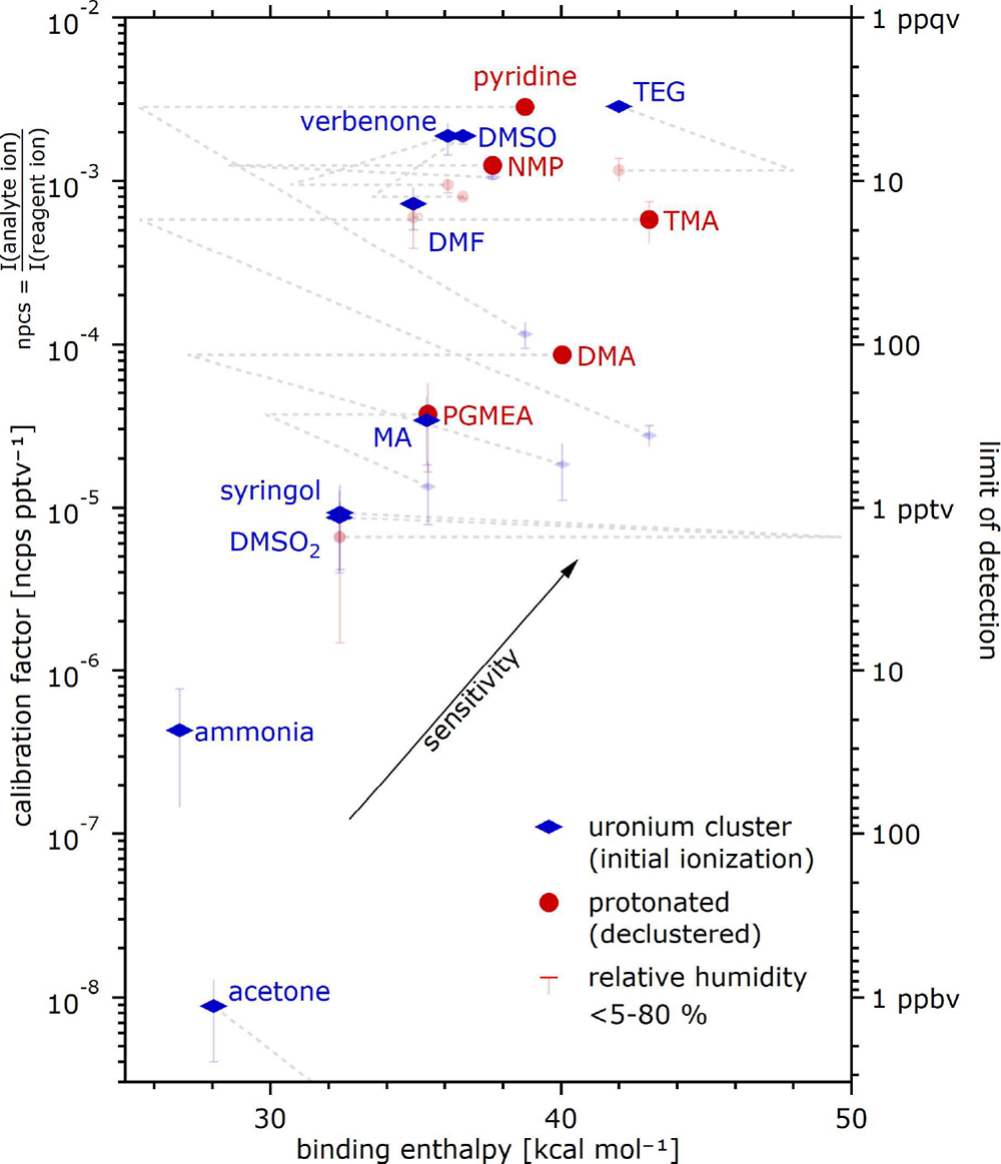
Sensitivity of uronium CIMS to different analytes. The left axis shows the calibration factors [ncps ppt^–1^], where ncps is the intensity of the product ion relative to the summed intensity of the reagent ions. The right axis illustrates estimated limits of detection, i.e., the VMR corresponding to a respective peak signal of 100 cps (not considering the instrument baseline). The abbreviations refer to the following compounds: DMSO: dimethyl sulfoxide; DMF: dimethylformamide; NMP: *N*-methyl-2-pyrrolidone; TEG: triethylene glycol; TMA: trimethylamine; DMA: dimethylamine; PGMEA: propylene glycol methyl ether acetate; MA: methylamine.

Figure 4 further shows the humidity sensitivity of the calibration factors, a known feature of different ionization schemes. In case of ammonia, one compound exhibiting a more pronounced sensitivity, the calibration factor varies by a factor 4.28, requiring proper accounting or controlling of humidity during measurements. Most of the other compounds tested exhibit a calibration factor that varies only weakly (median γ = 0.55; Table S2). While a clear trend is lacking in the limited set of compounds tested, the data are compatible with the idea that water helps stabilizing small newly formed ion–molecule clusters by providing additional vibrational modes and by sacrificial cooling, i.e., dissipating excess formation enthalpy by evaporating.^50^

Table S2 summarizes the experimental sensitivities. The detection limits attainable with uronium are in the parts per trillion by volume (pptv) to parts per quadrillion by volume (ppqv) range, as a combination of the favorable tendency of uronium to interact with the analytes and the maximized opportunity for ionization to occur during the relatively long reaction time (approximately 25 ms) under ambient pressure. For this reason, these detection limits are similar in magnitude to those attainable for sulfuric acid using a nitrate CIMS. It needs to be stressed that the calibration factors and associated limits of detection are directly influenced by the reaction time in the IMR and the IMR pressure and could be influenced by other factors such as instrument-dependent fragmentation.^51^ In absolute terms, at the maximum sensitivity, as few as 10 attomoles are required to produce 100 counts, the lowest detectable signal.

The sensitivity to dimethyl sulfide (DMSO) was characterized to be very high, with respective detection limits in the ppqv range. This is particularly interesting in light of the importance of atmospheric DMS oxidation, which by forming sulfuric acid is a globally important driver of particle formation. The details of the oxidation mechanism, e.g., the branching of pathways, are still being investigated.^52,53^ Beyond DMSO, clusters of uronium with DMSO2 (dimethyl sulfone) were apparently also observed in exploratory flow tube experiments of DMS oxidation.

The sensitivity to amines is likewise favorably high, for detection both as clusters with uronium and as protonated molecules. In our oxidizing atmosphere that promotes the formation of acids, amines are important to atmospheric aerosol particle formation.^54,55^ Ammonia is only detected as a cluster with uronium, due to the mass of ammonium being too small for sensitive detection in the mass spectrometer. The amines (larger than methylamine) are also detected in their protonated form.

### Ambient NH_3_ Quantification

Figure 5 demonstrates the remarkable potential of uronium CIMS to quantify even low abundances of ammonia in atmospheric measurements. Here, lacking a suitable NH_3_-free scrubber that would allow rigorously characterizing the instrument background, the lowest ambient normalized signal intensity during the measurement period was used as an approximation for the instrument background. The background-corrected intensity of the detected peak was scaled by a humidity-dependent calibration factor (8.6 × 10^–7^ ncps pptv^–1^ for the moist ambient measurements and 6.2 × 10^–7^ ncps pptv^–1^ for drier indoor measurements). The baseline-corrected and scaled time series (1 min averages) are shown in Figure 5 (red markers), along with the QC-TILDAS time series. VMRs ranged from a few tens of pptv to single ppbv during the ambient measurements, to 3 ppbv indoor VMR and peaking at around 100 ppbv in the spiked experiment.

**Figure 5:**
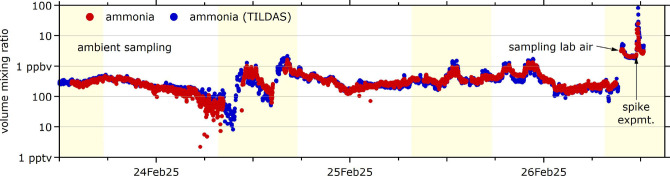
Ammonia abundance in ambient and laboratory air. Ammonia was evaporated into the laboratory on Feb 26 to determine the response to high VMR. The uronium CIMS-derived ammonia time series agrees well with the QC-TILDAS-determined time series.

The agreement between uronium CIMS and QC-TILDAS is excellent (standard deviation $\sigma \left(\frac{{\mathrm{NH}}_{3}\left(\mathrm{CIMS}\right)}{{\mathrm{NH}}_{3}\left(\mathrm{TILDAS}\right)}\right)=34\%$ ). The fairly long CIMS inlet line used for the comparison limits the temporal response to changes in the NH_3_ abundance. Differences in line conditioning hamper the direct comparison of the data during the transients of the laboratory experiment, but it appears that there is a good correlation over at least 2 orders of magnitude in abundance, from several tens of pptv to several ppbv. Determining the NH_3_ instrument baseline with a suitable ammonia scrubber would be required for the accurate determination of absolute ammonia concentrations in independent deployments of a uronium CIMS that lack the luxury of a reference instrument. NH_3_ is notorious for requiring rigorous characterization of the instrument background, reflected in the fact that QC-TILDAS utilizes 30% of the time assessing the instrument baseline, zeroing every 15 min. However, the excellent agreement for the given approach highlights that uronium CIMS can be used to measure atmospheric bases.

Uronium CIMS detects amines even more sensitively than ammonia, and calibration factors are less susceptible to humidity changes (Figure 4). The capability to quantify amine abundances is therefore in practice limited by the control over the instrument baseline and inlet effects. While multiple approaches to measure amines have been developed over the years,^56–66^ operational complexities of these approaches have hampered their wide adoption by the general atmospheric research community. We believe that the straightforward operation of the uronium CIMS may facilitate the routine measurements of amines, thereby providing a handle on constraining atmospheric particle formation.

### CI Scheme Complementarity

Figure 6 shows the capability of uronium to ionize organic molecules in the α-pinene oxidation experiments relative to nitrate and fluoranthenium ionization. Nitrate CI is well-known to be selective to highly oxygenated compounds.^67^ Low-pressure positive ionization (e.g., PTR) creates sensitivity to less oxygenated or nonoxygenated volatile compounds. Here, fluoranthenium was used to detect α-pinene, the nonoxidized precursor, and other reaction products.^7^ Uronium positive-mode ionization at ambient pressure bridges the gap between these two schemes and extends the sensitivity to oxygenated compounds. Figure 6 reinforces the fact that the chemical variety of trace gases requires the use of different ionization schemes across polarities and pressures to attain comprehensive detection sensitivity.

**Figure 6:**
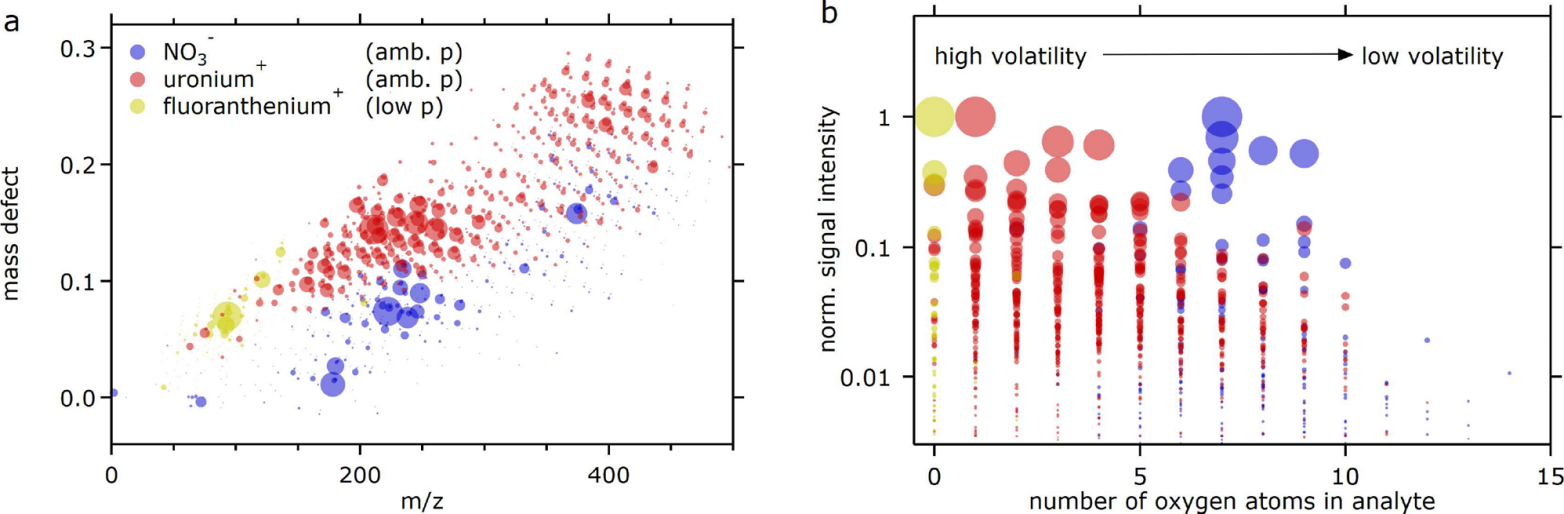
(a) Mass defect figure (corrected for reagent ion mass). (b) Intensities for compounds with varying numbers of oxygen atoms detected during α-pinene oxidation experiments using nitrate, uronium, and fluoranthenium ionization (blue, red, and yellow markers, respectively). The marker area is proportional to the normalized intensity in each mode. Among the multiple ionization schemes needed to ionize the spectrum of compounds, uronium creates sensitivity to moderately oxygenated, basic and polar neutral compounds.

## Conclusions

In this study, we introduced two novel approaches: X-ray desorption of solids at room temperature and uronium CI for the selective, ultrasensitive analysis of complex gas mixtures. First, we demonstrated uronium generation through X-ray desorption of solid urea, as well as nitrate from ammonium nitrate. Next, we studied the ionization mechanism of uronium and neutral molecules by using quantum-chemical computations. To confirm this empirically, we conducted multipoint calibrations across a broad range of humidities. We validated ambient quantification capabilities with side-by-side QC-TILDAS measurements of trace ammonia. To demonstrate the synergy of uronium with established CI schemes, we oxidized α-pinene and DMS and analyzed the products by multipressure CIMS.

The ability of uronium to selectively form strong clusters creates excellent sensitivities to several key compound classes. The exceptional sensitivity of uronium CI to basic compounds, including ammonia and amines—molecules that are notoriously difficult to measure—facilitates the enhanced and more routine assessment of bases in ambient CIMS deployments. The sensitivity range synergizes with that of low-pressure positive and ambient-pressure negative ionization, enabling a near-complete coverage with just one mass spectrometer.^6,7^

Importantly, using solid urea as a reagent precursor eliminates the need for hazardous chemicals, such as ammonia and amines, significantly enhancing operational safety and sustainability. The negligible vapor pressure of urea or ammonium nitrate prevents reagent contamination inside the instrument, enabling low consumption and maintenance-free operation over extended periods, demonstrated continuously for several months, with the potential to operate much longer. Given the minimal consumption of the solid reagent precursor, the routine use of isotopically labeled reagents becomes more feasible.

Together, these features advance CIMS safety, robustness, sustainability, and analytical performance, paving the way for standardization and wider industrial adoption. Future studies may explore reagent solids beyond urea and ammonium nitrate, as well as practical applications in fields ranging from atmospheric chemistry and environmental monitoring to industrial emission control and trace-gas detection in challenging environments.

## Supplementary Material



## Notes

Description of quantum-chemical calculations, calculated binding enthalpies, vapor pressure extrapolations, example of calibration experiment, calibration results, and detection limits (PDF)
