# Supplementary material for: Uronium from X‑ray-Desorbed Urea Enables Sustainable Ultrasensitive Detection of Amines and Semivolatiles
Source: Anal Chem. 2025 Sep 28;97(39):21282–90. doi: 10.1021/acs.analchem.5c02239 (PMC12509187; doi:10.1021/acs.analchem.5c02239)
Supplement: Supplementary file 1 [file ac5c02239_si_001.pdf]

Supplementary Information to: Uronium from X-ray Desorbed Urea Enables Sustainable Ultrasensitive Detection of Amines and Semivolatiles

Aleksei Shcherbinin,[*,†,‖] Henning Finkenzeller,[*,†,‡,‖] Fariba Partovi,[†] Netta Vinkvist,[§] Jussi Kontro,[†] Matthew Boyer,[‡] Joona Mikkilä,[†] Siddharth Iyer,[°] Jyri Mikkilä,[†] Paxton Juuti,[†] Nina Sarnela,[‡] Juha Kangasluoma,[‡] and Matti Rissanen[°,§]

[†]Karsa Ltd., Helsinki, Finland
[‡]Institute for Atmospheric and Earth System Research / Department of Physics, Faculty of Science, University of Helsinki, Helsinki, Finland
[°]Aerosol Physics Laboratory, Physics Unit, Faculty of Engineering and Natural Sciences, Tampere University, Tampere, Finland
[§]Department of Chemistry, Faculty of Science, University of Helsinki, Helsinki, Finland
[‖]author with equal contributions

E-mail: Aleksei.Shcherbinin@Karsa.fi; Henning.Finkenzeller@Helsinki.fi

Contents

List of Figures S2

1 Calibration experiment example S3

2 Vapor pressure of urea and ammonium nitrate S5

3 Description of quantum chemical calculations S5

4 Results of quantum chemical calculations S6

5 Experimental calibration factors and detection limits S9

References S11

List of Figures

S1 Calibration experiment example (pyridine, C_5H_5N): Time series. S3

S2 Calibration experiment example (pyridine, C_5H_5N): Normalized intensity as function of relative humidity, for different dosing ratios. S4

S3 Calibration experiment example (pyridine, C_5H_5N): Normalized intensity as function of dosed volume mixing ratio (VMR), for different humidities. S4

S4 Calibration experiment example (pyridine, C_5H_5N): Calibration factor as function of relative humidity. S4

S5 Saturation abundance of urea and ammonium nitrate in measurements and extrapolated to room temperature. S5

S6 Calculated binding enthalpies for the formation of analyte–uronium clusters from neutral analyte and uronium for compounds of different classes. S6

S7 Calculated binding enthalpies for the formation of analyte–uronium clusters for compounds of different classes, including declustering. S8

S8 Detection limits for different compounds either as cluster with uronium or protonated. S10

1 Calibration experiment example

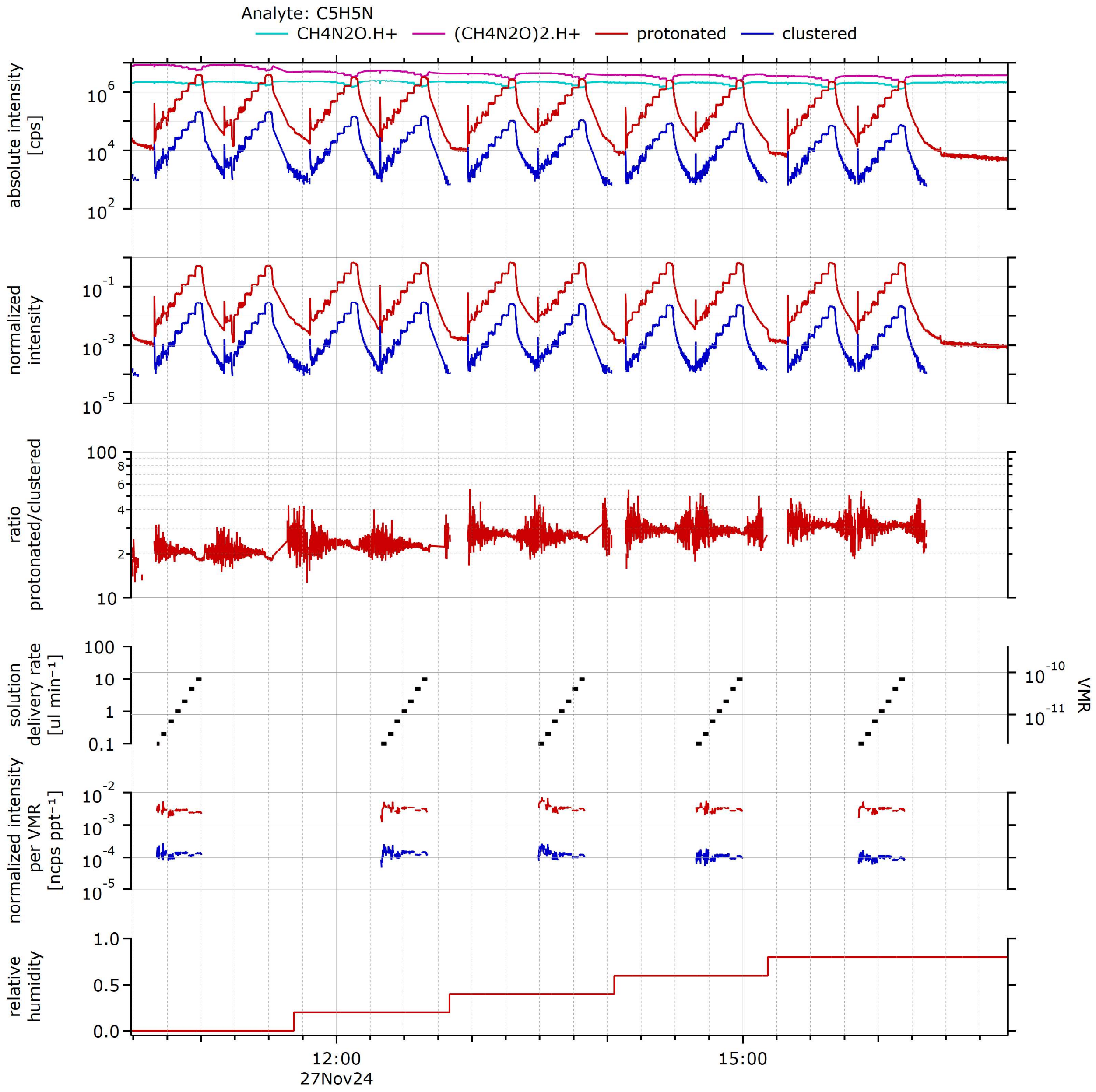

Figure S1: Calibration experiment example (pyridine, C_5H_5N): Time series of the raw intensities, normalized intensity, ratio of protonated and cluster signal, delivery rate for solution to liquid calibration unit, ratio of normalized intensity to volume mixing ratio (VMR), and relative humidity (ranging from $< 5\%$ to 80%). Here, the normalization was performed relative to the sum of uronium monomer and dimer.

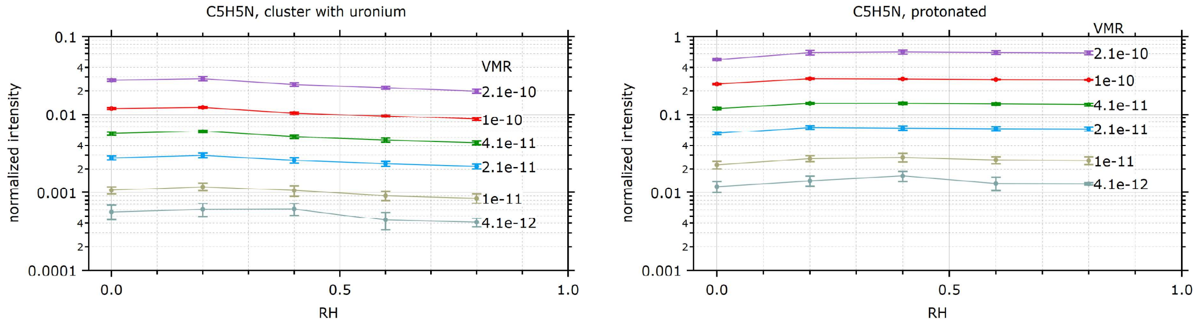

Figure S2: Calibration experiment example (pyridine, C_5H_5N): Normalized intensity as function of relative humidity, for different dosing ratios.

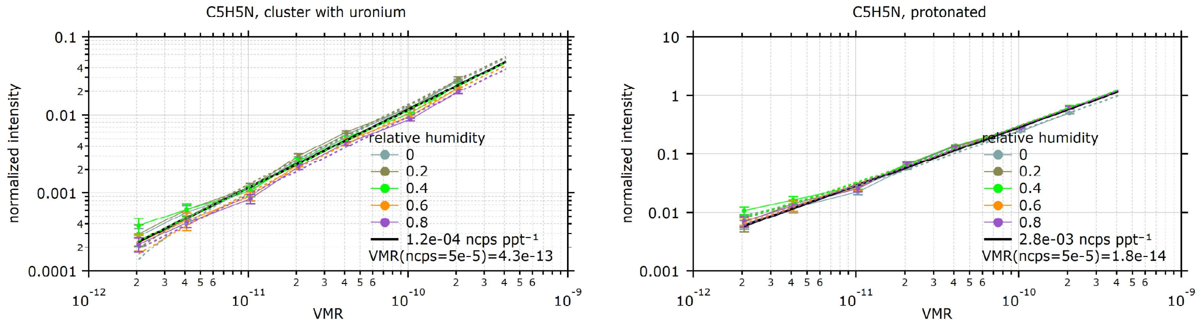

Figure S3: Calibration experiment example (pyridine, C_5H_5N): Normalized intensity as function of dosed volume mixing ratio (VMR), for different humidities.

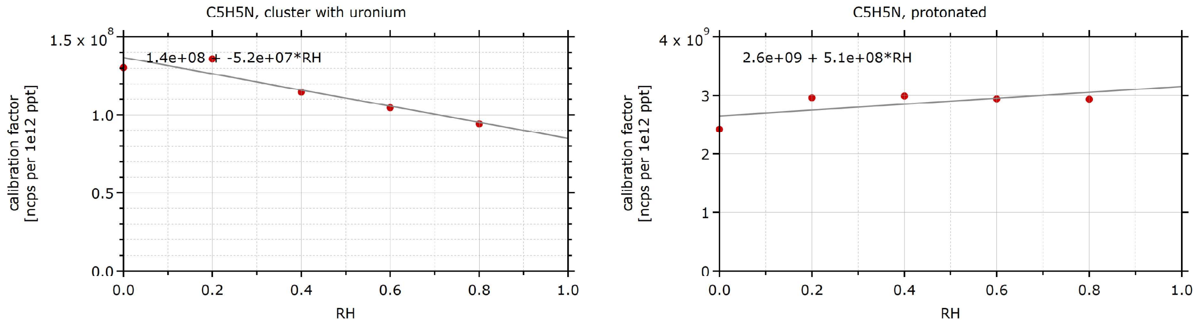

Figure S4: Calibration experiment example (pyridine, C_5H_5N): Calibration factor as function of relative humidity.

2 Vapor pressure of urea and ammonium nitrate

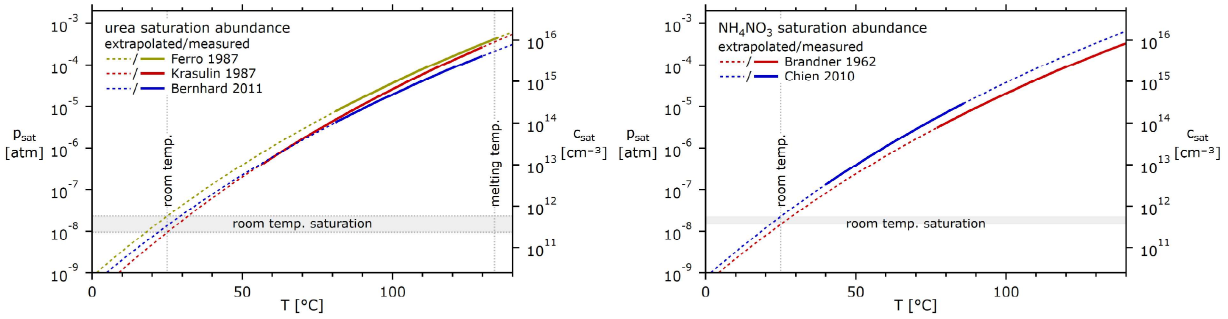

Figure S5: Saturation abundance of urea and ammonium nitrate in measurements and extrapolated to room temperature.[1–5]

3 Description of quantum chemical calculations

A systematic conformational search was done using the MMFF molecular mechanics method in the Spartan '24 program (Wavefunction, Inc). Single-point energies were computed at the B3LYP/6-31+G* level[6–8] for all conformers using Spartan '24, and those within 5 kcal mol^{-1} in electronic energies of the lowest-energy conformer were considered for geometry optimizations. Geometry optimizations were first carried out at the B3LYP/6-31+G* level of theory and subsequently at ωB97X-D/6-31+G* (with frequency calculations)[9] for the conformers within 2 kcal mol^{-1} in electronic energies of the lowest-energy conformer. The energies of the lowest energy conformers were further refined at the DLPNO-CCSD(T)/def2-QZVPP level of theory. These geometry optimizations and frequency calculations were performed with the Gaussian 16 program,[10] while the ORCA 5.0.3 program[11] was used for DLPNO-CCSD(T) calculations.

As the hydrogen bonding sites are less obvious for the iodine-containing systems, a different approach was adopted to find their lowest energy clusters with uronium. The partial atomic charges for all iodine molecules and uronium were calculated with Gaussian 16 using the Pop=MKUFF keyword at the M062X/aug-cc-pVTZ-(PP) level of theory. Iodine pseudo-potentials were taken from the EMSL basis set library.[12,13] The atomic charges were then used for conformational sampling where the molecules were treated as rigid (intramolecular bonds and angles between bonds remain fixed) using the Artificial Bee Colony (ABC) algorithm implemented in the ABCluster program.[14–16] The following values were used for key parameters: pop = 1000, gen = 100, -lm = 3000. The resulting structures were then first optimized using semi-empirical xTB method with "vTight" criteria.[17–19] The structures were then filtered to remove duplicates, as described in Kubecka et al.[15] The resulting structures were first optimized at the M062X/SDD level of theory, and then at the M062X/aug-cc-pVTZ-PP level of theory on the subset of molecules within 5 kcal mol^{-1} of the lowest energy geometry in relative electronic energies. Finally, the energies of the lowest energy molecules were refined at the DLPNO-CCSD(T)/def2-QZVPP level of theory using ORCA program, which were used to calculate the cluster binding enthalpies.

4 Results of quantum chemical calculations

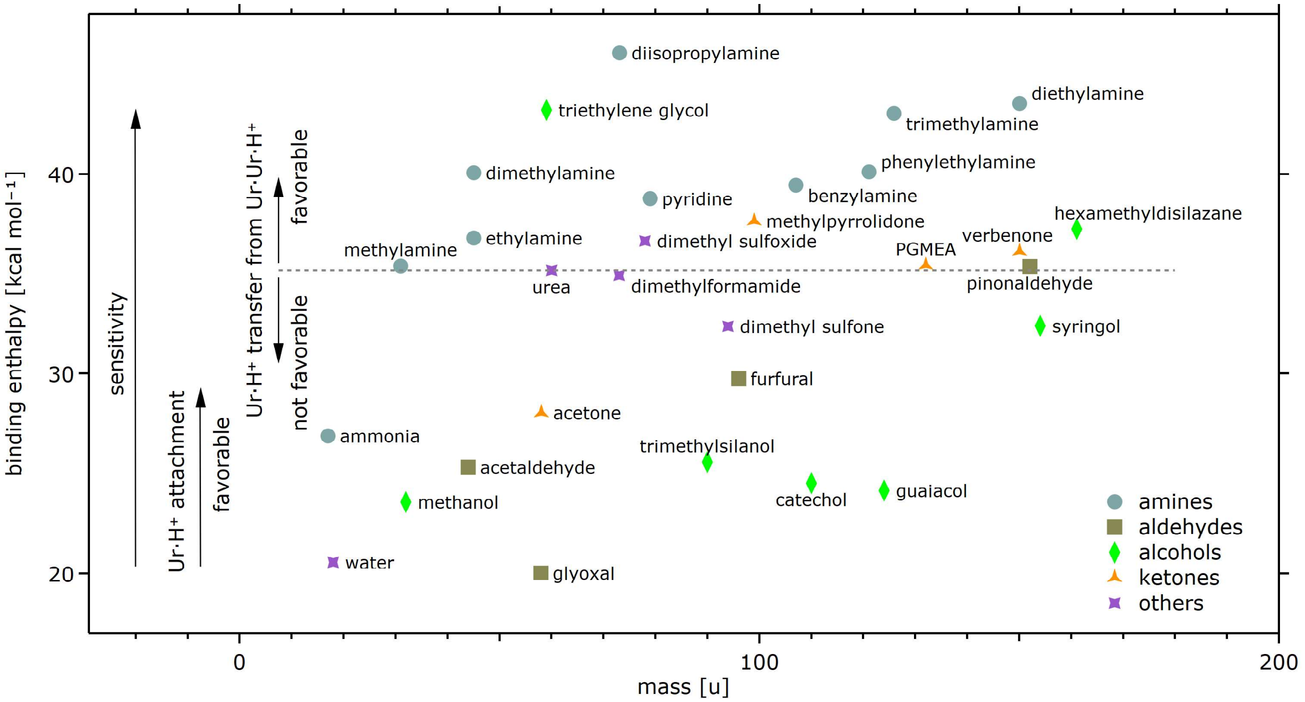

Figure S6: Calculated binding enthalpies for the formation of analyte–uronium clusters from neutral analyte and uronium for compounds of different classes. Uronium transfer from the protonated urea-dimer ion is energetically favorable for enthalpies larger than 35.16 kcal mol^{-1}. The abbreviations refer to the following compounds: Ur: urea; PGMEA: propylene glycol methyl ether acetate; DMSeO: dimethyl selenoxide.

Table S1: Calculated enthalpies for the declustering of the target–uronium cluster ($A \cdot Ur \cdot H^+$) into analyte (A) and uronium ($Ur \cdot H^+$)), and protonated analyte ($A \cdot H^+$) and urea (Ur).

target	sum formula	$AUrH^+ \rightarrow A + UrH^+$ kcal mol^{-1}	$AUrH^+ \rightarrow AH^+ + Ur$ kcal mol^{-1}
acetaldehyde	C_2H_4O	25.32	50.44
catechol	$C_6H_6O_2$	24.52	52.20
dimethylformamide	C_3H_7NO	34.91	35.24
dimethyl sulfoxide	C_2H_6SO	36.63	33.51
dimethyl sulfone	$C_2H_6SO_2$	32.37	49.45
furfural	$C_5H_4O_2$	29.75	37.11
guaiacol	$C_7H_8O_2$	24.14	48.65
pinonealdehyde	$C_{10}H_{16}O$	35.36	28.97
pyridine	C_5H_5N	38.76	25.44
syringol	$C_8H_{10}O_3$	32.40	49.63
verbenone	$C_{10}H_{14}O$	36.11	30.70
urea	CH_4N_2O	35.16	35.16
ammonia	NH_3	26.88	32.51
benzylamine	C_7H_9N	39.44	27.89
diethylamine	$C_4H_{11}N$	43.53	25.31
diisopropylamine	$C_6H_{15}N$	46.06	23.80
dimethylamine	C_2H_6NH	40.05	27.15
water	H_2O	20.56	64.91
methanol	CH_3OH	23.57	52.48
methylamine	CH_3NH_2	35.38	29.84
phenylethylamine	$C_8H_{11}N$	40.11	26.22
trimethylamine	C_3H_9N	43.03	25.51
acetone	C_3H_6O	28.04	43.06
ethylamine	C_2H_6NH	36.79	28.08
PGMEA[a]	$C_6H_{12}O_3$	35.42	29.83
trimethylsilanol	$C_3H_{10}OSi$	25.56	41.59
hexamethyldisiloxane	$C_6H_{18}OSi_2$	37.23	21.25
glyoxal	$C_2H_2O_2$	20.01	64.47
methylpyrrolidone	C_5H_9NO	37.65	28.56
iodine	I_2	10.32	
iodine oxide	IO	22.52	
iodine dioxide	OIO	21.59	
diiodine dioxide	I_2O_2	33.47	
diiodine trioxide	I_2O_3	29.54	
diiodine tetroxide	I_2O_4	35.51	
diiodine pentoxide	$IOIO_4$	25.90	
hypoiodous oxide	HOI	19.54	
iodous oxide	HIO_2	33.80	
iodic oxide	HIO_3	27.44	

a: propylene glycol methyl ether acetate

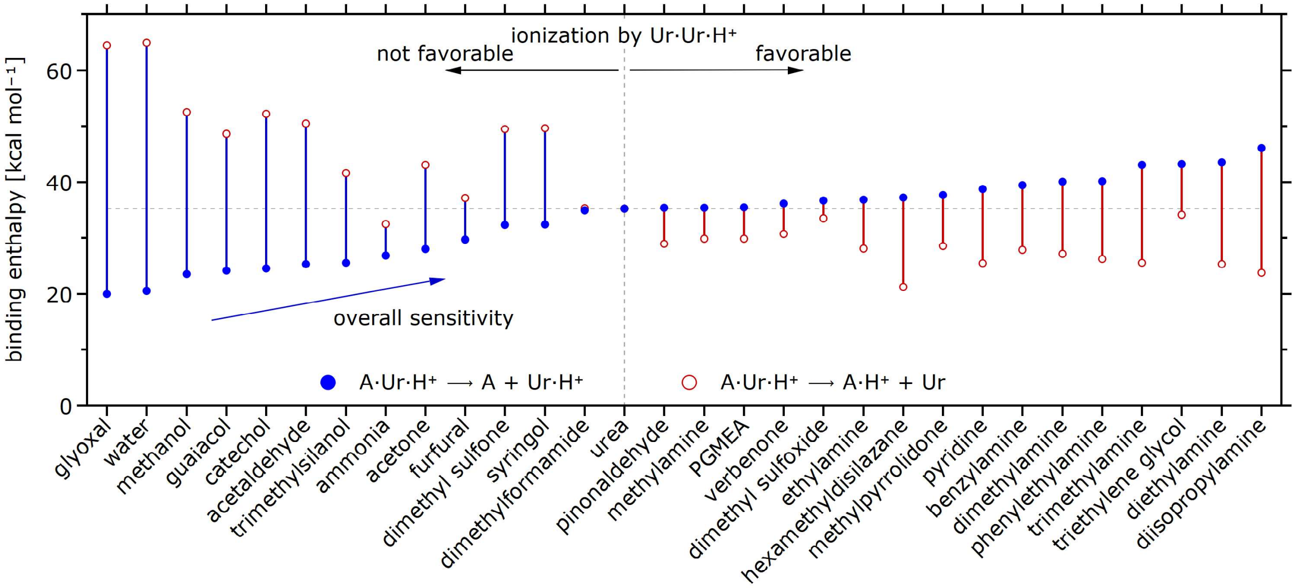

Figure S7: Calculated binding enthalpies for the formation of analyte–uronium clusters for compounds, including also the enthalpy of declustering of the analyte–uronium cluster into protonated analyte and neutral urea (hollow red markers). The abbreviations refer to the following compounds: Ur: urea; PGMEA: propylene glycol methyl ether acetate.

5 Experimental calibration factors and detection limits

Table S2: Experimental calibration factors c and detection limits λ for different compounds. Here, c_0 is the calibration factor under dry conditions, γ the coefficient of relative humidity sensitivity, such that for a given humidity $\phi \in [0, 1]$ the calibration factor is $c = c_0(1+\gamma\phi)$ [ncps pptv^{-1}]. λ_{100} [pptv] is the detection limit equivalent to 100 cps signal, the approximately lowest signals still detectable. λ_{BG} [pptv] is the detection limit equivalent to the instrument background (if present*), indicating the magnitude of background correction that may be needed. λ_{3SDEV} [pptv] is the detection limit that corresponds to three times the standard deviation of a the instrument background (if present).

		cluster					protonated				
target	sum formula	c_0	γ	λ_{100}	λ_{BG}	λ_{3SDEV}	c_0	γ	λ_{100}	λ_{BG}	λ_{3SDEV}
ammonia	NH_3	1.5E-7	4.28	23	2300	350					
MA	CH_3NH_2	4.8E-5	-0.62	0.29	0.78						
DMA	$(CH_3)_2NH$	2.5E-5	-0.55	0.54	1.4	0.88	8.2E-5	0.12	0.12	0.19	0.051
TMA	$(CH_3)_3N$	2.4E-5	0.33	0.36	1.0	0.21	4.2E-4	0.79	0.017		
pyridine	C_5H_5N	1.4E-4	-0.30	0.086	0.59	0.45	2.6E-3	0.15	0.0035	0.48	0.10
DMF	C_3H_7NO	9.1E-4	-0.45	0.014	0.86	0.19	7.8E-4	-0.50	0.017	0.91	0.32
DMSO	C_2H_6OS	2.1E-3	-0.19	0.0053	0.023	0.0057	8.2E-4	-0.05	0.012	0.027	0.012
DMSO2	$C_2H_6O_2S$	1.3E-5	-0.69	1.1			1.3E-5	-1.05	1.5		
syringol	$C_8H_{10}O_3$	1.4E-5	-0.70	1.1	2.5	2.7	1.1E-5	-0.86	1.5	5.6	1.7
verbenone	$C_{10}H_{14}O$	2.3E-3	-0.36	0.0053	0.013	0.0051	1.0E-3	-0.18	0.011		
PGMEA	$C_6H_{12}O_3$	1.9E-5	-0.59	0.74	1.9		5.8E-5	-0.71	0.27		
acetone	C_3H_6O	1.3E-8	-0.69	1100	2800	3800	9.3E-11	-0.40	130000		
TEG	$C_6H_{14}O_4$	2.8E-3	0.05	0.0035	0.083	0.026	9.9E-4	0.39	0.0085	0.10	0.053
NMP	C_5H_9NO	1.1E-3	-0.07	0.0094	0.09	0.044	1.3E-3	-0.05	0.008	0.11	0.043

*Orbitrap mass spectrometers typically only report positive detections of a peak above a certain intensity threshold[20] to avoid excessively large spectra files.

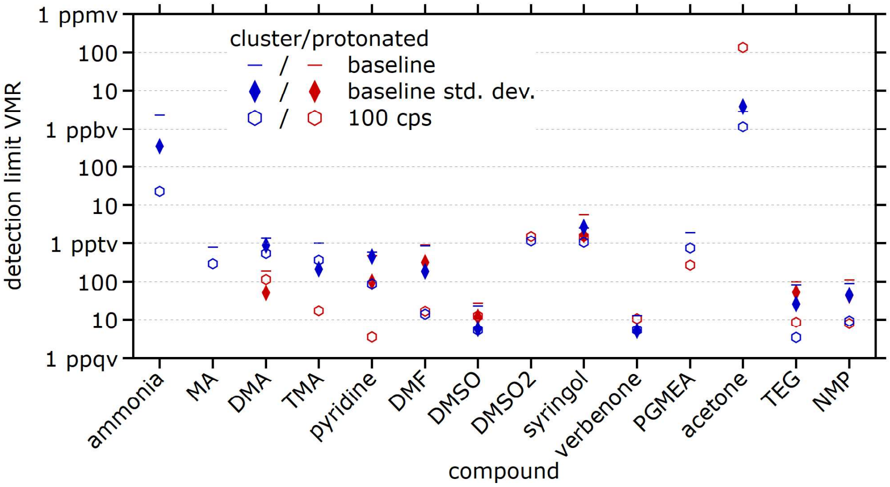

Figure S8: Detection limits for different compounds either as cluster with uronium or protonated. Detection limits for different compounds either as cluster with uronium (blue markers) or protonated (red markers). The detection limits are estimated in different ways: First, the horizontal lines indicate is the volume mixing ratio equivalent to the instrument background (if present) when the inlet is overflowed with pure nitrogen. The diamond marker indicate the detection limit calculated as three times the standard deviation of a the instrument background (if present). Lastly, the hexagonal marker is the volume mixing ratio equivalent to 100 cps signal, the approximately lowest signals still detectable.

References

(1) Ferro, D.; Barone, G.; Della Gatta, G.; Piacente, V. Vapour pressures and sublimation enthalpies of urea and some of its derivatives. *The Journal of Chemical Thermodynamics* **1987**, *19*, 915–923.

(2) Krasulin, A. P.; Kozyro, A. A.; Kabo, G. Y. Saturation vapor pressure of urea in the temperature range 329-403 K. **1987**, *60*, 96–99.

(3) Bernhard, A. M.; Czekaj, I.; Elsener, M.; Wokaun, A.; Kröcher, O. Evaporation of Urea at Atmospheric Pressure. *The Journal of Physical Chemistry A* **2011**, *115*, 2581–2589.

(4) Brandner, J. D.; Junk, N. M.; Lawrence, J. W.; Robins, J. Vapor Pressure of Ammonium Nitrate. *Journal of Chemical & Engineering Data* **1962**, *7*, 227–228.

(5) Chien, W.-M.; Chandra, D.; Lau, K. H.; Hildenbrand, D. L.; Helmy, A. M. The vaporization of NH4NO3. *The Journal of Chemical Thermodynamics* **2010**, *42*, 846–851.

(6) Becke, A. D. Density-functional thermochemistry. III. The role of exact exchange. *The Journal of Chemical Physics* **1993**, *98*, 5648–5652.

(7) Lee, C.; Yang, W.; Parr, R. G. Development of the Colle-Salvetti correlation-energy formula into a functional of the electron density. *Physical Review B* **1988**, *37*, 785–789.

(8) Frisch, M. J.; Pople, J. A.; Binkley, J. S. Self-consistent molecular orbital methods 25. Supplementary functions for Gaussian basis sets. *The Journal of Chemical Physics* **1984**, *80*, 3265–3269.

(9) Chai, J.-D.; Head-Gordon, M. Long-range corrected hybrid density functionals with damped atom–atom dispersion corrections. *Physical Chemistry Chemical Physics* **2008**, *10*, 6615–6620.

(10) Frisch, M. J. et al. Gaussian 16 Revision C.01. 2016.

(11) Neese, F.; Wennmohs, F.; Becker, U.; Riplinger, C. The ORCA quantum chemistry program package. *The Journal of Chemical Physics* **2020**, *152*, 224108.

(12) Kendall, R. A.; Dunning Jr., T. H.; Harrison, R. J. Electron affinities of the first-row atoms revisited. Systematic basis sets and wave functions. *The Journal of Chemical Physics* **1992**, *96*, 6796–6806.

(13) Dunning Jr., T. H. Gaussian basis sets for use in correlated molecular calculations. I. The atoms boron through neon and hydrogen. *The Journal of Chemical Physics* **1989**, *90*, 1007–1023.

(14) Kubečka, J.; Besel, V.; Neefjes, I.; Knattrup, Y.; Kurtén, T.; Vehkamäki, H.; Elm, J. Computational Tools for Handling Molecular Clusters: Configurational Sampling, Storage, Analysis, and Machine Learning. *ACS Omega* **2023**, *8*, 45115–45128.

(15) Kubečka, J.; Besel, V.; Kurtén, T.; Myllys, N.; Vehkamäki, H. Configurational Sampling of Noncovalent (Atmospheric) Molecular Clusters: Sulfuric Acid and Guanidine. *The Journal of Physical Chemistry A* **2019**, *123*, 6022–6033.

(16) Zhang, J.; Dolg, M. ABCluster: the artificial bee colony algorithm for cluster global optimization. *Phys. Chem. Chem. Phys.* **2015**, *17*, 24173–24181.

(17) Bannwarth, C.; Caldeweyher, E.; Ehlert, S.; Hansen, A.; Pracht, P.; Seibert, J.; Spicher, S.; Grimme, S. Extended tight-binding quantum chemistry methods. *WIREs Computational Molecular Science* **2021**, *11*, e1493.

(18) Bannwarth, C.; Ehlert, S.; Grimme, S. GFN2-xTB—An Accurate and Broadly Parametrized Self-Consistent Tight-Binding Quantum Chemical Method with Multipole Electrostatics and Density-Dependent Dispersion Contributions. *Journal of Chemical Theory and Computation* **2019**, *15*, 1652–1671.

(19) Pracht, P.; Caldeweyher, E.; Ehlert, S.; Grimme, S. A robust non-self-consistent tight-binding quantum chemistry method for large molecules. **2019**,

(20) Cai, R.; Huang, W.; Meder, M.; Bourgain, F.; Aizikov, K.; Riva, M.; Bianchi, F.; Ehn, M. Improving the Sensitivity of Fourier Transform Mass Spectrometer (Orbitrap) for Online Measurements of Atmospheric Vapors. *Analytical Chemistry* **2022**, *94*, 15746–15753.
